# Association of NTproBNP and cTnI with outpatient sudden cardiac death in hemodialysis patients: the Choices for Healthy Outcomes in Caring for ESRD (CHOICE) study

**DOI:** 10.1186/s12882-016-0230-x

**Published:** 2016-02-20

**Authors:** Rachel M. Kruzan, Charles A. Herzog, Aozhou Wu, Yingying Sang, Rulan S. Parekh, Kunihiro Matsushita, Seungyoung Hwang, Alan Cheng, Josef Coresh, Neil R. Powe, Tariq Shafi

**Affiliations:** Department of Medicine, Johns Hopkins University, Baltimore, MD USA; Department of Medicine, Division of Cardiology, Hennepin County Medical Center, University of Minnesota, Minneapolis, MN USA; Department of Epidemiology, Johns Hopkins Bloomberg School of Public Health, Baltimore,, MD USA; Department of Biostatistics, Johns Hopkins Bloomberg School of Public Health, Baltimore, MD USA; Departments of Medicine and Pediatrics, University of Toronto, Toronto, Canada; Welch Center for Prevention, Epidemiology and Clinical Research, Johns Hopkins University, Baltimore, MD USA; Department of Medicine, Division of Cardiology, Johns Hopkins University, Baltimore, MD USA; Department of Medicine, University of California, San Francisco, CA USA; Department of Medicine, Division of Nephrology, Johns Hopkins University, 301 Mason Lord Drive, Suite, 2500 Baltimore, MD USA

**Keywords:** Sudden Cardiac Death, Hemodialysis, NTproBNP, Troponin I

## Abstract

**Background:**

Sudden cardiac death (SCD) is the most common etiology of death in hemodialysis patients but not much is known about its risk factors. The goal of our study was to determine the association and risk prediction of SCD by serum N-terminal prohormone of brain natriuretic peptide (NTproBNP) troponin I (cTnI) in hemodialysis patients.

**Methods:**

We measured NTproBNP and cTnI in 503 hemodialysis patients of a national prospective cohort study. We determined their association with SCD using Cox regression, adjusting for demographics, co-morbidities, and clinical factors and risk prediction using C-statistic and Net Reclassification Improvement (NRI).

**Results:**

Patients’ mean age was 58 years and 54 % were male. During follow-up (median 3.5 years), there were 75 outpatient SCD events. In unadjusted and fully-adjusted models, NTproBNP had a significant association with the risk of SCD. Analyzed as a continuous variable, the risk of SCD increased 27 % with each 2-fold increase in NTproBNP (HR, 1.27 per doubling; 95 % CI, 1.13–1.43; *p* < 0.001). In categorical models, the risk of SCD was 3-fold higher in the highest tertile of NTproBNP (>7,350 pg/mL) compared with the lowest tertile (<1,710 pg/mL; HR for the highest tertile, 3.03; 95 % CI, 1.56–5.89; *p* = 0.001). Higher cTnI showed a trend towards increased risk of SCD in fully adjusted models, but was not statistically significant (HR, 1.17 per doubling; 95 % CI, 0.98–1.40; *p* = 0.08). Sensitivity analyses using competing risk models showed similar results. Improvement in risk prediction by adding cardiac biomarkers to conventional risk factors was greater with NTproBNP (C-statistic for 3-year risk: 0.810; 95 % CI, 0.757 to 0.864; and continuous NRI: 0.270; 95 % CI, 0.046 to 0.495) than with cTnI.

**Conclusions:**

NTproBNP is associated with the risk of SCD in hemodialysis patients. Further research is needed to determine if biomarkers measurement can guide SCD risk prevention strategies in dialysis patients.

**Electronic supplementary material:**

The online version of this article (doi:10.1186/s12882-016-0230-x) contains supplementary material, which is available to authorized users.

## Background

At least one-quarter of deaths in dialysis patients are estimated to be from sudden cardiac death (SCD) [1]. This increased risk of SCD in dialysis patients is likely affected by the multiple, associated co-morbid conditions, such as diabetes [2] and hypertension [3], left ventricular hypertrophy [4], ischemic heart disease [5], inflammation [6] and perhaps the dialysis treatment itself with intermittent fluid and electrolyte fluctuations [2].

A large gap of knowledge still remains as to which dialysis patients are at the highest risk of suffering SCD. Cardiac biomarkers may identify patients with subclinical cardiovascular disease that are at increased risk for SCD. The N-Terminal fragment of the pro-hormone brain natriuretic peptide (NTproBNP) is a marker of myocardial stretch and volume overload [7]. Serum cardiac troponin I (cTnI) is a marker of cardiac damage. Elevated cTnI levels in dialysis patients are associated with increased risk of all-cause and cardiovascular mortality, but few studies have investigated the association between these biomarkers and SCD in hemodialysis patients [8, 9].

The aim of our study was to determine if elevated levels of NTproBNP and cTnI are associated with increased risk for SCD in hemodialysis patients. We also evaluated whether these cardiac makers improve the risk prediction of SCD beyond conventional predictors in hemodialysis patients.

## Methods

### Study design

The Choices for Healthy Outcomes in Caring for ESRD (CHOICE) Study is a longitudinal, prospective cohort study of 1041 patients starting dialysis that were recruited from 81 dialysis centers in 19 states. Eligibility criteria included dialysis initiation in preceding 3 months, age 18 years or older, ability to speak English or Spanish, and ability to consent. Each participant completed informed consent. The participants were enrolled from October 1995 to June 1998 and were on average 45 days post-dialysis initiation. A specimen bank was established for all Dialysis Clinic, Inc. (DCI) participants. Our analysis included 503 hemodialysis participants with available stored specimens. The Johns Hopkins Medicine Institutional Review Board and the clinical centers’ review boards approved the study and all participants provided informed consent.

### Cardiac biomarkers (Serum NTproBNP and cTnI)

We collected blood samples prior to routine outpatient dialysis session, centrifuged within 30–45 min of collection, and sent them overnight on ice to the central laboratory. We divided each sample into multiple vials and stored at −80 °C till they were thawed and aliquoted for this study. We measured NT-pro-BNP using a one-step sandwich chemiluminescent immunoassay also based on LOCI* technology. We measured cTnI by homogeneous, sandwich chemiluminescent immunoassay based on LOCI* technology. Both NTproBNP and cTnI were measured on the Dimension Vista System at the University of Maryland School of Medicine, Baltimore, Maryland. The coefficient of variation (CV) for NT-pro-BNP was 5.0 % at 107.2 pg/mL and 1.6 % at 275 pg/mL and 1.2 % at 3,313 pg/mL. The reliability correlation coefficient for NT-pro-BNP was 0.998. The CV for cTnI was 9.1 % at 0.090 ng/mL, 5.9 % at 1.08 ng/mL and 1.6 % at 4.87 ng/mL. The reliability correlation coefficient for cTnI in a 5 % sample of masked duplicate specimens was 0.969.

### Outcome

The primary outcome for this study was outpatient SCD defined as an out-of-hospital deaths including deaths that occurred in an emergency department or one in which the patient was reported to be “dead on arrival” with the following codes noted in the National Death Index death certificate data: ICD-9 390–398, 402 or 404–429; and ICD-10, I00-I09, I11, I13 and I20–I51, as previously described [6]. We excluded deaths with hyperkalemia, sepsis or malignancy listed as a contributing cause of death or if the death occurred while under hospice care.

### Other covariates

We collected data on participants’ age, sex, race and body mass index (BMI). We adjudicated baseline comorbidities including prevalent cardiovascular disease and left ventricular hypertrophy (as assessed by electrocardiograms) by abstraction of dialysis unit records, hospital discharge summaries, medication lists, consultation notes, diagnostic imaging, and cardiac imaging reports and Index of Coexistent Disease (ICED) scoring. For this index, comorbid conditions, including diabetes mellitus, ischemic heart disease, congestive heart failure, hypertension, peripheral vascular disease, and other conditions, were determined present by two trained nurses, and then, the severity of each comorbid condition for each patient was calculated using Index of Disease Severity (IDS) and Index of Physical Impairment (IPI) (30). These two scores were then used to calculate the ICED score, which serves as a validated medical record-derived index that captures both presence and severity of comorbid conditions^19,20,30^. ICED scores range from 0 to 3, with 3 as the highest severity level. We abstracted antihypertensive medication use at baseline from patients’ charts and obtained routine laboratory data from medical records. We measured serum albumin (CV, 1.9 %) in the same specimen as NTproBNP and cTnI at University of Minnesota, Minneapolis, Minnesota. We also used data on C-reactive protein, interleukin-6 and p-selectin that had been previously measured for research [10, 11]. Other laboratory data measured in routine clinical care included hemoglobin, calcium, phosphate, blood urea nitrogen, creatinine, glucose, potassium and bicarbonate. These laboratory data were obtained from the same time as cardiac biomarker assessment.

### Statistical analysis

We compared the baseline characteristics of the participants across categories of NTproBNP and cTnI using chi-squared test for categorical variables and t-tests for continuous variables. Missing data for variables were as follows: educational status (2.8 %), smoking history (2.8 %), BMI (5.6 %) systolic blood pressure (3.8 %), serum potassium, calcium and phosphate (6.8 %) and serum bicarbonate (12 %). Missing data values were imputed with 10 data replicates using multiple imputation by the chained equations method implemented by the ice program in Stata. We modeled NTproBNP and cTnI as continuous variables after natural log transformation and as categorical variables. We categorized NTproBNP as tertiles and cTnI as a low, mid and high category. The low cTnI group included those with cTnI below detection limit; (<0.015 ng/mL; *n* = 336). Remaining patients are divided into two groups at the median with the mid category referring to group with detectable values but below median (<0.040 ng/mL; *n* = 85) and high category referring to the group with values at or above median (≥0.040 ng/mL; *n* = 82). We visualized the association between NTproBNP and cTnI and outcomes by calculating incidence rates for SCD adjusted for age, sex and race using a Poisson regression model with biomarkers modeled as linear spline with 2 knots corresponding to the categories. We used Cox proportional hazards regression to analyze the association between the biomarkers and SCD. We assessed proportional hazards assumptions graphically and by tests of Schoenfeld residuals. We used hazard ratios (HRs) to quantify the associations of biomarkers with SCD after adjustment for a priori defined confounders, including demographic characteristics [age, sex, race (white or other)] and clinical factors [smoking status (ever versus never), ICED score, diabetes, cardiovascular disease, left ventricular hypertrophy, congestive heart failure, BMI and systolic blood pressure], laboratory tests (hemoglobin, serum albumin, serum potassium, serum bicarbonate, serum corrected calcium and serum phosphate) and β-blocker use. We conducted sensitivity analyses further adjusting for biomarkers previously associated with SCD in CHOICE study (i.e., CRP, IL-6, and p-selectin) and accounting for the competing risk of death from other causes using competing-risks regression based on Fine and Gray’s proportional subhazards model. In exploratory analyses we further determined SCD risk association within subgroups by testing for interactions with age, sex, race, cardiovascular disease, diabetes, serum albumin, serum potassium and serum bicarbonate, with continuous variables categorized above or below median. We evaluated risk prediction by NTproBNP and cTnI over 5-years by calculating C statistic and net reclassification improvement (NRI) [12, 13]. We assessed model calibration using modified Hosmer–Lemeshow statistic [14, 15]. We performed all statistical analyses using Stata software, version 12.1 (Stata Corp.). We defined statistical significance as p <0.05 using two-tailed tests.

## Results

### Baseline characteristics

The final study sample comprised of 503 hemodialysis patients. Compared to the overall cohort, the included patients were less likely to be White and had higher urea creatinine, potassium, calcium, phosphate and albumin and lower hemoglobin and Kt/Vurea (Additional file 1: Table S1). The average age of the participants was 58 years and 54 % were men. At baseline, 57 % of the participants had diabetes, 56 % had cardiovascular disease, and 24 % had a history of myocardial infarction. Table 1 summarizes the baseline characteristics of the participants according to categories of NTproBNP and cTnI. Patients with higher NTproBNP and cTnI were older, more likely to have a history of cardiovascular disease, and have elevated IL-6 levels. In addition, patients with higher NTproBNP were more likely to be White, had lower body BMI and higher CRP whereas those with higher cTnI were more likely to be male.

**Table 1:** Characteristics of 503 Hemodialysis Patients by Levels of NTproBNP and cTnI

		NTproBNP (pg/mL) Categories				Troponin I (ng/mL) Categories			
						Below Detection Limit	Detectable		
Characteristic	Overall	Low	Mid	High	*p*	Low	Mid	High	*p*
Range, minimum to maximum		59–1710	1728–7269	7350–273502	-	<0.015	0.015–0.039	0.04–3.09	-
N (%)	503 (100)	168 (33.4)	168 (33.4)	167 (33.2)	-	336 (66.8)	85 (16.9)	82 (16.3)	-
TNI, median (25^th^-75^th^ percentiles) ng/mL	<0.015(<0.015–0.023)	<0.015	<0.015(<0.015–0.020)	0.021(<0.015–0.049)	-	<0.015	0.023(0.020–0.029)	0.085(0.052–0.162)	-
NTproBNP, median (25^th^-75^th^ percentiles) pg/mL	3138(1233–9835)	822.5(489.5–1238)	3145.5(2364–4622)	14735(9835–24240)	-	2205(945–5670.5)	7166(2326–16018)	10242(4187–23407)	-
Demographics									
Age, years	57.8 (14.7)	52.7 (13.8)	59.8 (15.2)	60.9 (13.6)	<0.001	56.1 (15.4)	60.3 (13.1)	62.1 (11.9)	<0.001
White	321 (63.8)	99 (58.9)	101 (60.1)	121 (72.5)	0.017	225 (67.0)	50 (58.8)	46 (56.1)	0.107
Male	273 (54.3)	91 (54.2)	88 (52.4)	94 (56.3)	0.773	164 (48.8)	55 (64.7)	54 (65.9)	0.002
Clinical Characteristics									
Body Mass Index, Kg/m^2^	27.4 (7.1)	28.2 (7.8)	28.1 (7.5)	25.8 (5.6)	0.002	27.6 (7.3)	27.0 (6.7)	26.9 (6.9)	0.297
Cause of End Stage Renal Disease									
Diabetes mellitus	248 (49.3)	77 (45.8)	82 (48.8)	89 (53.3)	0.389	157 (46.7)	46 (54.1)	45 (54.9)	0.259
Hypertension	86 (17.1)	21 (12.5)	27 (16.1)	38 (22.8)	0.041	50 (14.9)	18 (21.2)	18 (22.0)	0.172
Glomerulonephritis	74 (14.7)	36 (21.4)	25 (14.9)	13 (7.8)	0.002	58 (17.3)	9 (10.6)	7 (8.5)	0.068
Other	95 (18.9)	34 (20.2)	34 (20.2)	27 (16.2)	0.547	71 (21.1)	12 (14.1)	12 (14.6)	0.189
ICED = 3	147 (29.3)	41 (24.4)	46 (27.5)	60 (35.9)	0.057	97 (29.0)	20 (23.5)	30 (36.6)	0.175
Diabetes	287 (57.2)	85 (50.6)	95 (56.9)	107 (64.1)	0.045	180 (53.7)	54 (63.5)	53 (64.6)	0.087
Cardiovascular Disease	279 (55.6)	64 (38.1)	102 (61.1)	113 (67.7)	<0.001	165 (49.3)	58 (68.2)	56 (68.3)	<0.001
Congestive Heart Failure	247 (49.2)	58 (34.5)	80 (47.9)	109 (65.3)	<0.001	146 (43.6)	54 (63.5)	47 (57.3)	0.001
Coronary Heart Disease	209 (41.6)	42 (25)	77 (46.1)	90 (53.9)	<0.001	114 (34.0)	47 (55.3)	48 (58.5)	<0.001
Myocardial Infarction	120 (23.9)	20 (11.9)	51 (30.5)	49 (29.3)	<0.001	68 (20.3)	23 (27.1)	29 (35.4)	0.012
Left Ventricular Hypertrophy	137 (27.3)	43 (25.6)	41 (24.6)	53 (31.7)	0.281	74 (22.1)	29 (34.1)	34 (41.5)	0.001
Time Since Start of Dialysis, Months	5.0 (1.6)	5.0 (1.5)	4.7 (1.5)	5.2 (1.6)	0.189	4.9 (1.5)	5.3 (1.5)	5.1 (1.7)	0.168
Laboratory Tests									
Blood Urea Nitrogen, mg/dL	57.8 (15.1)	57.6 (15.3)	58.3 (15.4)	57.6 (14.8)	0.998	57.3 (15.5)	59.3 (14.9)	58.4 (14.1)	0.482
Kt/V_UREA_	1.3 (0.302)	1.3 (0.324)	1.3 (0.278)	1.3 (0.303)	0.250	1.3 (0.316)	1.3 (0.255)	1.3 (0.292)	0.788
Creatinine, mg/dL	8.0 (2.9)	8.2 (2.8)	8.2 (3.2)	7.6 (2.5)	0.081	8.1 (2.9)	7.9 (2.7)	7.6 (2.8)	0.311
Potassium, mEq/L	4.7 (0.665)	4.6 (0.584)	4.7 (0.712)	4.7 (0.680)	0.036	4.7 (0.642)	4.7 (0.689)	4.7 (0.733)	0.665
Glucose, mg/dL	167.0 (103.0)	162.3 (79.0)	164.0 (82.7)	174.8 (137.2)	0.780	159.9 (83.4)	178.4 (120.0)	183.9 (145.3)	0.222
Bicarbonate, mEq/L	20.4 (2.9)	20.7 (2.7)	20.0 (3.0)	20.5 (2.9)	0.510	20.4 (2.9)	20.7 (2.5)	20.2 (3.4)	0.919
Hemoglobin, g/dL	11.0 (1.3)	11.2 (1.5)	10.9 (1.3)	10.8 (1.3)	0.003	11.0 (1.4)	11.2 (1.2)	10.8 (1.4)	0.686
Corrected Calcium, mg/dL	9.8 (0.883)	9.7 (0.854)	9.8 (0.954)	9.8 (0.839)	0.471	9.8 (0.881)	9.8 (0.918)	9.6 (0.844)	0.050
Phosphate, mg/dL	5.5 (1.7)	5.5 (1.6)	5.5 (1.8)	5.6 (1.6)	0.348	5.5 (1.7)	5.8 (1.6)	5.6 (1.9)	0.283
Albumin, g/dL	3.5 (0.549)	3.7 (0.503)	3.5 (0.568)	3.4 (0.547)	<0.001	3.5 (0.533)	3.5 (0.621)	3.5 (0.538)	0.559
CRP, mg/L (median, 25^th^ – 75^th^ percentiles)	0.445(0.203–1.219)	0.382(0.158–0.779)	0.486(0.224–1.317)	0.51 (0.217–1.496)	0.007	0.441(0.195–1.229)	0.431(0.148–1.04)	0.489(0.222–1.155)	0.980
IL-6, pg/mL (median, 25^th^ – 75^th^ percentiles)	4.8 (2.8–8.2)	3.8 (2.3–5.6)	5.2 (2.7–8.9)	6.2 (3.6–11.5)	<0.001	4.3 (2.6–7.4)	5.3 (3.4–9.0)	6.1 (3.6–11.7)	0.008
P-Selectin, ng/ml (median, 25^th^ – 75^th^ percentiles)	97.2 (75.6–126.0)	93.4 (74.0–125.9)	96(73.9–124.1)	98.7 (79.4–130.2)	0.526	96.3 (76.2–127.7)	94.7(72.2–117.0)	105.5(78.2–124.5)	0.627
Antihypertensive Medications, %									
β-blockers	123 (24.5)	36 (21.4)	40 (23.8)	47 (28.1)	0.350	84 (25.0)	21 (24.7)	18 (22.0)	0.846
ACE-inhibitors	147 (29.2)	40 (23.8)	51 (30.4)	56 (33.5)	0.136	87 (25.9)	29 (34.1)	31 (37.8)	0.058
Calcium Channel Blockers	309 (61.4)	105 (62.5)	109 (64.9)	95 (56.9)	0.304	205 (61.0)	60 (70.6)	44 (53.7)	0.077

Abbreviations: Troponin I, TNI; Hazard Ratio, HR; N-terminal pro-brain natriuretic peptide, NTproBNP

For TNI, low category refers those patients with TNI below the limit of detection (<0.015 ng/mL; *n* = 336). Remaining patients are divided into two groups at the median. Mid category refers to the group below median TNI for those with detectable values (<0.040 ng/mL; *n* = 85) and high category refers to those with values at or above median (≥0.040 ng/mL; *n* = 82)

For NTproBNP, low, mid and high category refers to the lowest, middle and highest tertiles of NTproBNP

*P*-values represent p-trend by linear regression for continuous variables and chi-square p-values for categorical variables

### Association of NTproBNP and cTnI with SCD

**Fig. 1:**
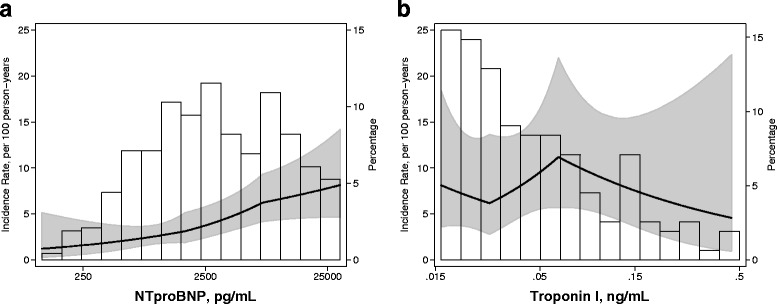
Adjusted incidence rate of sudden cardiac death in 503 participants of the CHOICE Study. Incidence rate per 100 person years adjusted for age, sex and race. Results are presented separately for (**a**) N-terminal pro-brain natriuretic peptide (NTproBNP) and (**b**) cardiac troponin I (cTnI). Line represents hazard ratio and shaded area represents the 95 % confidence interval. Vertical bars represent the distribution of the biomarkers. Data are limited to ≥0.5^th^ percentile and ≤99.5^th^ percentiles

The 75 SCD events occurred over 1,814 person-year of follow-up (median 3.5 years) with an incidence rate of 41.4 SCD events over 1000 person-years. The causes of death in these 75 patients, cross-tabulated by the causes listed in the National Death Index and Center for Medicare and Medicaid Services (CMS) death notification form (Form 2746) are presented in Additional file 1: Table S2. Fig. 1 presents the incidence rate of SCD adjusted for age, sex and race, demonstrating a linear increase in SCD incidence rate with NTproBNP and a relatively flat association with cTnI. In unadjusted models and minimally adjusted models, both NTproBNP and cTnI were associated with risk of SCD (Table 2). After adjustment for demographics, clinical factors, comorbidities, laboratory tests and β-blocker use, NTproBNP continued to have a statistically significant association [HR, 95 % confidence interval (CI)] with SCD [1.27 (1.13–1.43); *p* < 0.001]] whereas as the association between cTnI and SCD was not statistically significant [1.17 (0.98–1.40); *p* = 0.08]. Similar results were noted in the categorical analysis (Table 2). In the fully adjusted models, compared to the lowest tertile of NTproBNP, those in the highest tertile had a more than 3-fold higher risk of SCD [3.03 (1.56–5.89); *p* = 0.001]. Compared to the low cTnI category (undetectable cTnI), those in the mid (detectable and below median) and high cTnI category (detectable and above median) had a non-significant trend towards increased risk of SCD in the fully adjusted models.

**Table 2:** Association of NTproBNP and cTnI with Sudden Cardiac Death among 503 Hemodialysis Patients of the CHOICE Study

				Model 1		Model 2		Model 3		Model 4	
	Range	N (events)	Crude IR	HR (95 % CI)	*p*	HR (95 % CI)	*p*	HR (95 % CI)	*p*	HR (95 % CI)	*p*
NTproBNP, pg/mL											
Continuous ^a^		503 (75)		1.33 (1.21–1.46)	<0.001	1.29 (1.17–1.42)	<0.001	1.28 (1.14–1.44)	<0.001	1.27 (1.13–1.43)	<0.001
Categorical ^b, d^											
Low Category	59–1710	168 (13)	19.0	Reference		Reference		Reference		Reference	
Mid Category	1728–7269	168 (22)	35.3	1.99 (1.25–3.14)	0.003	1.99 (1.16–3.41)	0.012	1.67 (1.01–2.77)	0.045	1.39 (0.79–2.44)	0.252
High Category	7350–273502	167 (40)	78.9	4.49 (2.61–7.71)	<0.001	3.90 (2.07–7.34)	<0.001	3.32 (1.67–6.59)	0.001	3.03 (1.56–5.89)	0.001
*p-trend*				<0.001		<0.001		0.001		0.001	
Troponin I, ng/mL											
Continuous ^a^		503 (75)		1.19 (1.06–1.32)	0.002	1.22 (1.07–1.38)	0.002	1.18 (1.02–1.36)	0.027	1.17 (0.98–1.40)	0.084
Categorical ^b, c^											
Low Category	<0.015	336 (41)	32.8	Reference		Reference		Reference		Reference	
Mid Category	0.015–0.039	85 (18)	57.5	1.82 (1.06–3.10)	0.029	1.83 (1.04–3.20)	0.036	1.56 (0.88–2.80)	0.128	1.62 (0.89–2.95)	0.11
High Category	0.040–3.09	82 (16)	64.0	2.14 (1.46–3.13)	<0.001	2.48 (1.51–4.06)	<0.001	1.98 (1.15–3.39)	0.013	1.91 (0.98–3.72)	0.058
*p-trend*				<0.001		<0.001		0.011		0.048	

Abbreviations: Troponin I, cTnI; Hazard Ratio, HR; N-terminal pro-brain natriuretic peptide, NTproBNP. Incidence rate per 1000 person-years

^a^ Hazard ratio per doubling of the marker; modeled as ln(marker)/ln(2). Modeled using Cox proportional hazards regression

^b^ Hazard ratio with the low category as the reference group. Modeled using Cox proportional hazards regression

^c^ For cTnI, low category refers those patients with TNI below the limit of detection (<0.015 ng/mL; *n* = 336). Remaining patients are divided into two groups at the median. Mid category refers to the group below median cTnI for those with detectable values (<0.040 ng/mL; *n* = 85) and high category refers to those with values at or above median (≥0.040 ng/mL; *n* = 82)

^d^ For NTproBNP, low, mid and high category refers to the lowest, middle and highest tertiles of NTproBNP

Model 1: Unadjusted

Model 2: Adjusted for demographics including age, sex and race

Model 3: Adjusted for variables in model 2 + clinical factors including smoking status (ever versus never), Index of Coexistent Disease (ICED) score, diabetes, cardiovascular disease, congestive heart failure, body mass index and systolic blood pressure

Model 4: Adjusted for variables in model 3 + left ventricular hypertrophy, β-blocker use and laboratory tests including hemoglobin, serum albumin, serum potassium, serum bicarbonate, serum corrected calcium and serum phosphate

### Sensitivity analyses of the association between NTproBNP and cTNI and SCD

Further adjustment for markers of inflammation (CRP, IL6 and p-selectin) did not significantly change the magnitude or the direction of association (data not presented). Analyses using competing risks models showed results similar to the primary analysis (Additional file 1: Table S3).

### Exploratory analyses of the association between NTproBNP and cTnI and SCD

**Fig. 2:**
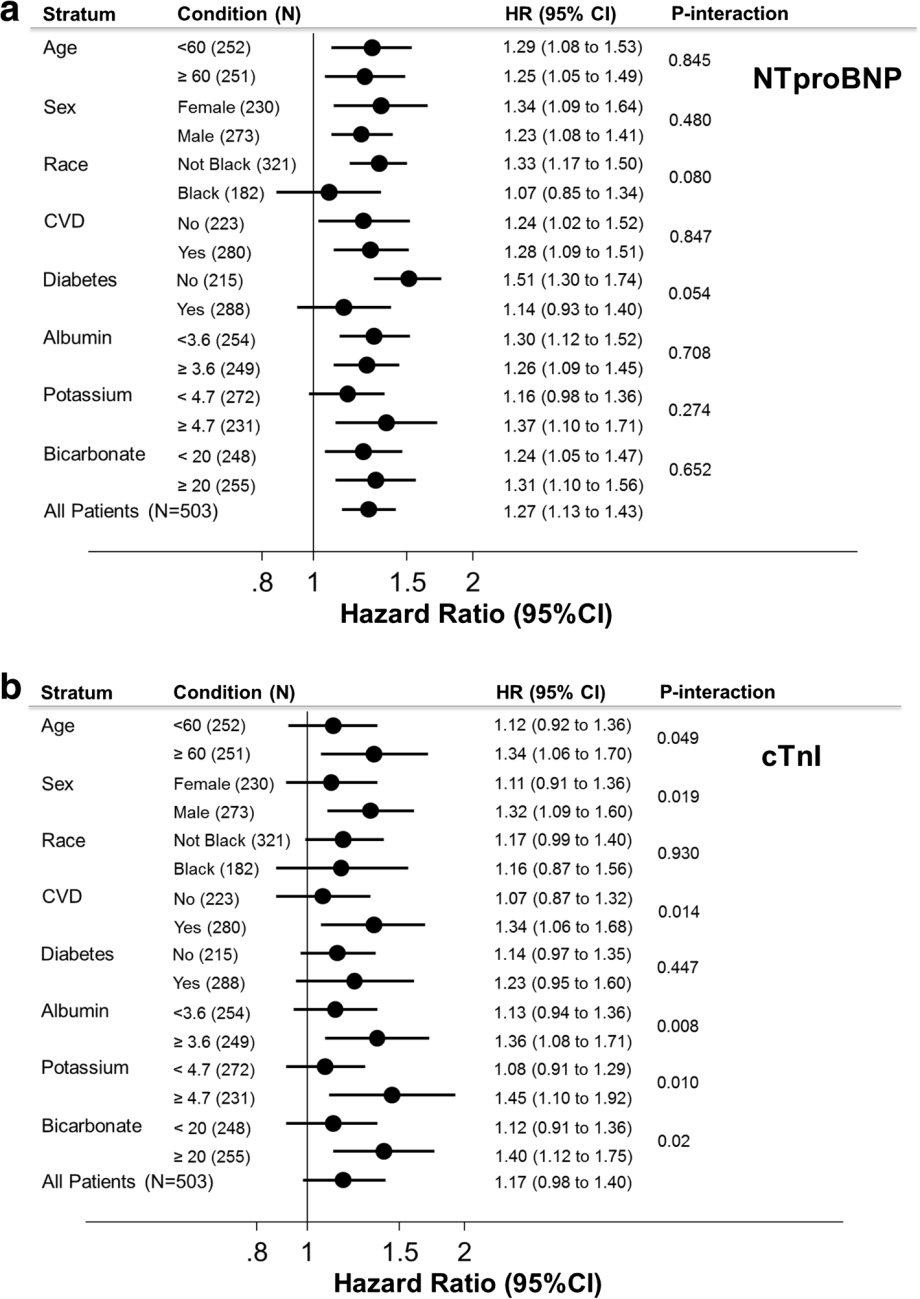
Subgroup analysis of the association between (**a**) NTproBNP and (**b**) cTnI and sudden cardiac death in 503 participants of the CHOICE Study. Hazard ratios are adjusted for variable in Model 4: demographics (age, sex and race), clinical factors [smoking status (ever versus never), Index of Coexistent Disease (ICED) score, diabetes, cardiovascular disease, congestive heart failure, body mass index and systolic blood pressure], left ventricular hypertrophy, β-blocker use and laboratory tests including hemoglobin, serum albumin, serum potassium, serum bicarbonate, serum corrected calcium and serum phosphate

The subgroup analyses should be interpreted with caution due to small sample size and multiple comparisons. There were no significant interactions in the models for NTproBNP (Fig. 2a) but there was suggestion of effect modification by cTnI and sex (p-interaction =0.02), baseline cardiovascular disease (p-interaction =0.01), serum albumin (p-interaction =0.008), serum potassium (p-interaction = 0.01) and bicarbonate (p-interaction = 0.02).

### Risk prediction of SCD with NTproBNP and cTnI

Compared to the fully adjusted model, the improvement in 3-year and 5-year risk prediction was greater with NTproBNP than cTnI (Table 3). Addition of both NTproBNP and cTnI to the model did not lead to further improvement in risk prediction.

**Table 3:** Risk Prediction for Sudden Cardiac Death with NTproBNP and cTnI in 503 Hemodialysis Patients of the CHOICE Study

	Model 4^a^	+NTproBNP	+cTnI	+NTproBNP and cTnI
3-Year Risk				
C-Statistic	0.790 (0.728 to 0.851)	0.810 (0.757 to 0.864)	0.791 (0.732 to 0.849)	0.810 (0.756 to 0.864)
Change in C-Statistic^b^	Ref	0.020 (−0.016 to 0.057)	0.001 (−0.019 to 0.021)	0.020 (−0.017 to 0.057)
NRI, Binary	Ref	0.125 (0.032 to 0.219)	0.033 (−0.021 to 0.088)	0.133 (0.039 to 0.227)
NRI, Continuous	Ref	0.262 (−0.048 to 0.572)	0.125 (−0.186 to 0.436)	0.212 (−0.098 to 0.522)
5-Year Risk				
C-Statistic	0.773 (0.723 to 0.823)	0.797 (0.751 to 0.843)	0.778 (0.730 to 0.827)	0.797 (0.751 to 0.843)
Change in C-Statistic	Ref	0.024 (−0.004 to 0.053)	0.006 (−0.009 to 0.021)	0.024 (−0.005 to 0.053)
NRI, Binary	Ref	0.063 (−0.028 to 0.154)	0.001 (−0.065 to 0.067)	0.072 (−0.020 to 0.164)
NRI, Continuous	Ref	0.270 (0.046 to 0.495)	0.018 (−0.207 to 0.243)	0.356 (0.132 to 0.581)

Abbreviations: *SCD* sudden cardiac death; NTproBNP, and N-terminal prohormone of brain natriuretic peptide; *cTnI* cardiac troponin I; *CVD* cardiovascular disease; *DM* diabetes mellitus; C-statistic, concordance statistic, *NRI* net reclassification index

^a^ Model 4: Adjusted for demographics (age, sex and race), clinical factors [smoking status (ever versus never), Index of Coexistent Disease (ICED) score, diabetes, cardiovascular disease, congestive heart failure, body mass index and systolic blood pressure], left ventricular hypertrophy, β-blocker use and laboratory tests including hemoglobin, serum albumin, serum potassium, serum bicarbonate, serum corrected calcium and serum phosphate

^b^ Change in C-statistic by adding biomarker to the variables in Model 4

## Discussion

In this national prospective cohort study of incident dialysis patients, we found a significant association between NTproBNP levels and the risk of outpatient SCD. Patients in the highest tertile of NTproBNP (>7,350 pg/mL) had a more than 3-fold higher risk of SCD compared with those in the lowest tertile (<1,710 pg/mL). NTproBNP also improved risk prediction of SCD with improvement in 3-year and 5-year NRI. Higher cTnI (>0.04 ng/mL) was also associated with a 91 % higher risk of SCD although it was of borderline statistical significance.

NTproBNP is a marker of myocardial stretch and correlates with reduced left ventricular function and volume overload in dialysis patients [3, 16, 17]. A number of previous studies have reported the association between elevated NTproBNP and cTnI and all-cause and cardiovascular mortality [18–20]. However, only a few prior studies have studied the association between these markers and SCD in dialysis patients. In the German Diabetes and Dialysis Study (4D-Study), NTproBNP above the fourth quartile (≥9,252 pg/mL) was associated with 2-fold higher risk of SCD compared with the lowest quartile. However, the study only included patients with diabetes. In a study of 230 peritoneal dialysis patients, NTproBNP but not troponin T was associated with an increased risk of SCD [3]. Our study extends these findings to a national cohort of US dialysis patients and shows a stronger association of NTproBNP compared with cTnI with SCD.

cTnI is an inhibitory protein within the troponin-tropomyosin complex that regulates striated muscle contraction [21]. The troponin complex is released in damaged cardiac muscle and serum levels are sensitive markers of cardiac injury [21, 22]. In our study, the findings of weaker association between cTnI and SCD compared with NTproBNP could reflect a greater risk of arrhythmia and SCD with volume overload and ventricular stretch detected by NTproBNP versus elevation of cTnI which could be from myocardial injury but could also reflect decreased renal clearance [23]. In previous dialysis studies, prevalence of elevated cTnI is lower than prevalence of elevated troponin T [18] and elevated cTnI levels have been inconsistently associated with increased risk of death. [23, 24] Whether these findings represent biological associations or the effect of assay variability at the low threshold will require future studies with comparison of different cTnI assay techniques, including the high-sensitivity troponin I assays [25].

In addition to evaluating NTproBNP and cTnI as a risk factor for SCD, we assessed its role in improving risk prediction. To our knowledge, prior studies of cardiac biomarkers in hemodialysis patients have not assessed improvement in risk prediction by cardiac biomarkers over traditional risk factors. In a study by Goldstein et al. using electronic medical record data, blood pressure, ultrafiltration and serum albumin predicted short-term (within a day of last outpatient hemodialysis) SCD in dialysis patients but cardiac biomarkers were not measured. [26] The rate of SCD in dialysis patients, 41 per 1000 person-years in our study, was substantially higher than that reported in the general population [27]. Implantable cardioverter-defibrillators (ICDs) can prevent SCD but are associated with a higher risk of infection [28, 29]. There are no randomized controlled data to guide primary prevention ICDs in this high risk population. Improvement in risk prediction of SCD by NTproBNP may help with identifying the patients at highest risk of SCD and facilitate their enrollment in future trials of SCD prevention.

There are some limitations to our study. First, we had a single measurement of biomarkers in stored specimens. The serum concentrations in individuals may change over time and the long storage time may effect serum concentrations. Second, our definition of SCD was based on location at time of death and the results may not apply to inpatient deaths many of which may be due to ventricular arrhythmias. The proportion of SCD was lower in our cohort (75/503; 15 %) versus that reported by USRDS (26.5 %). This could be due to difference in SCD definition (Additional file 1: Table S4) or due to relatively healthier patients in our cohort that survived to ~6 months when blood samples were obtained. Exclusion of inpatient deaths is likely to underestimate the risk. Third, patients with available samples may have been in better health compared to the overall cohort as they had higher baseline serum albumin and creatinine than included patients. It is possible that the associations may have been even stronger in the full cohort. Finally, even though we adjusted for a number of confounders, the possibility of uncontrolled confounding remains from factors, such as echo or magnetic resonance imaging based left ventricular mass and dialysate composition, which were not available in this study. These limitations are counterbalanced by our prospective study design with detailed assessments of baseline comorbidities complete and extensive information on covariates and inflammatory markers which are not available on most registry based retrospective dialysis cohorts. The use of NDI data rather than Form 2746 data is also an important strength of this study. Form 2746 is filled out by the primary nephrologist in the dialysis unit, sometimes weeks after death, and they may not know of the cause of death. NDI uses death certificate data which is from providers who were closer to patients’ care at the time of death. This approach reduces the risk of misclassification of outcomes (Additional file 1: Table S2).

## Conclusions

In conclusion, our study demonstrates a significant association between NTproBNP and SCD and suggests that elevated NTproBNP level in dialysis patients is a potent predictor for SCD. SCD is a potentially modifiable cause of death in dialysis patients. Risk-centered approaches have the potential to identify patients at highest risk for SCD and test therapies for its prevention.

## Additional file

Additional file 1:
**Supplemental Tables 1–4.** (DOCX 21 kb)

## Abbreviations

SCD: sudden cardiac death
NTproBNP: N-Terminal fragment of the prohormone brain natriuretic peptide
cTnI: cardiac troponin I
CHOICE: Choices for Healthy Outcomes in Caring for ESRD
DCI: Dialysis Clinic, Inc.
CV: coefficient of variation
ICED: Index of Coexistent Disease
BMI: Body mass index
NRI: net reclassification improvement
CMS: Center for Medicare and Medicaid Services
HR: hazard ratio
CI: confidence interval
