# Supplementary material for: Association of NTproBNP and cTnI with outpatient sudden cardiac death in hemodialysis patients: the Choices for Healthy Outcomes in Caring for ESRD (CHOICE) study
Source: BMC Nephrol. 2016 Feb 20;17:18. doi: 10.1186/s12882-016-0230-x (PMC4761195; doi:10.1186/s12882-016-0230-x)
Supplement: Additional file 1: — Supplemental Tables 1–4. (DOCX 21 kb) [file 12882_2016_230_MOESM1_ESM.docx]

**Supplemental Table 1: Comparison of the Baseline Characteristics of the Included versus Excluded Participants***

| **Characteristic** | **Available Samples**  **(Included)** | **No Samples**  **(Excluded)** | **P (Included vs. Excluded)** |
| --- | --- | --- | --- |
| **Numbers** | 503 | 538 |  |
| **Demographics** |  |  |  |
| Age, years | 57.8 (14.7) | 58.0 (15.2) | 0.842 |
| White | 321 (63.8) | 425 (79.0) | <0.001 |
| Male | 273 (54.3) | 291 (54.1) | 0.952 |
|  |  |  |  |
| **Clinical Characteristics** |  |  |  |
| Body Mass Index, Kg/m^2^ | 27.4 (7.1) | 26.8 (6.3) | 0.197 |
| Cause of End Stage Renal Disease |  |  | 0.32 |
| Diabetes mellitus | 248 (49.3) | 233 (43.8) |  |
| Hypertension | 86 (17.1) | 94 (17.7) |  |
| Glomerulonephritis | 74 (14.7) | 94 (17.7) |  |
| Other | 95 (18.9) | 110 (20.7) |  |
| ICED=3 | 147 (29.3) | 161 (30.0) | 0.805 |
| Diabetes | 287 (57.2) | 274 (51.0) | 0.047 |
| Cardiovascular Disease | 279 (55.6) | 314 (58.5) | 0.346 |
| Congestive Heart Failure | 247 (49.2) | 239 (44.5) | 0.129 |
| Coronary Heart Disease | 209 (41.6) | 248 (46.2) | 0.140 |
| Myocardial Infarction | 120 (23.9) | 135 (25.1) | 0.644 |
| Left Ventricular Hypertrophy | 137 (27.3) | 122 (22.7) | 0.089 |
|  |  |  |  |
| **Laboratory Tests** |  |  |  |
| Blood Urea Nitrogen, mg/dL | 58.0 (13.5) | 55.6 (15.6) | 0.03 |
| Kt/V_UREA_ | 1.2 (0.3) | 1.5 (0.5) | <0.001 |
| Creatinine, mg/dL | 7.9 (2.5) | 7.2 (2.6) | <0.001 |
| Potassium, mEq/L | 4.6 (0.6) | 4.4 (0.6) | <0.001 |
| Glucose, mg/dL | 167.7 (75.4) | 163.2 (80.7) | 0.355 |
| Hemoglobin, g/dL | 10.8 (1.0) | 11.1 (1.2) | <0.001 |
| Corrected Calcium, mg/dL | 9.5 (0.58) | 9.4 (0.60) | 0.003 |
| Phosphate, mg/dL | 5.5 (1.3) | 5.1 (1.2) | <0.001 |
| Albumin, g/dL | 3.7 (0.3) | 3.6 (0.4) | <0.001 |

Note: Numbers presented are mean (standard deviation) or percent unless otherwise specified.

Conversion factors for units: albumin in g/dL to g/L, x 10; calcium in mg/dL to mmol/L, x 0.2495; phosphate in mg/dL to mmol/L, x 0.3229; hemoglobin in g/dL to g/L, x 10; BUN in mg/dL to urea in mmol/L, x 0.357; creatinine in mg/dL to umol/L, x 88.4; No conversion is necessary for potassium in mEq/L to mmol/L.

P-values are by Student t-test for continuous variables and chi-square test for categorical variables

Abbreviations: Kt/V_UREA_: dialysis dose (K-dialyzer clearance of urea, t-dialysis time, V-volume of distribution of urea)

* Laboratory data in this table are baseline values from the first 3 months after enrollment in CHOICE Study and may differ from Table 1.

**Supplemental Table 2: Comparison of Cause of Death Assigned by National Death Index versus Center for Medicare and Medicaid Service (CMS) Death Notification Form (Form 2746)**

|  | **National Death Index Cause of Death** | | | | | | | | | |
| --- | --- | --- | --- | --- | --- | --- | --- | --- | --- | --- |
| **Form 2746**  **Cause of Death** | **MI** | | **Cardiomyopathy** | | **Arrhythmia** | | **Other** | | **Total** | |
|  | **SCD** | **Non-SCD** | **SCD** | **Non-SCD** | **SCD** | **Non-SCD** | **SCD** | **Non-SCD** | **SCD** | **Non-SCD** |
| **CVD/MI** | 6 (25%) | 9 (36%) | 4 (27%) | 6 (29%) | 3 (9%) | 8 (17%) | 0 | 13 (9%) | 13 (17%) | 36 (15%) |
| **Cardiac Arrest** | 10 (42%) | 5 (20%) | 3 (20%) | 7 (33%) | 13 (38%) | 17 (36%) | 1 (50%) | 33 (22%) | 27 (36%) | 62 (25%) |
| **Arrhythmia** | 2 (8%) | 3 (12%) | 1 (7%) | 0 | 4 (12%) | 6 (13%) | 0 | 5 (3%) | 7 (9%) | 14 (6%) |
| **Cardiomyopathy** | 0 | 0 | 2 (13%) | 4 (19%) | 0 | 1 (2%) | 1 (50%) | 4 (3%) | 3 (4%) | 9 (4%) |
| **Other Cardiac** | 0 | 0 | 1 (7%) | 0 | 1 (3%) | 2 (4%) | 0 | 1 (0.7%) | 2 (3%) | 3 (1%) |
| **Hyperkalemia** | 0 | 0 | 0 | 0 | 1 (3%) | 1 (2%) | 0 | 3 (2%) | 1 (1%) | 4 (2%) |
| **Infection** | 2 (8%) | 0 | 0 | 1 (5%) | 0 | 3 (6%) | 0 | 16 (11%) | 2 (3%) | 20 (8%) |
| **other** | 4 (17%) | 8 (32%) | 4 (27%) | 3 (14%) | 12 (35%) | 9 (19%) | 0 | 78 (51%) | 20 (27%) | 98 (40%) |
| **Total** | 24 (100%) | 25 (100%) | 15 (100%) | 21 (100%) | 34 (100%) | 47 (100%) | 2 (100%) | 153 (100%) | 75 (100%) | 246 (100%) |

Abbreviations: CVD, atherosclerotic cardiovascular disease; MI, myocardial infarction; SCD, sudden cardiac death

**Supplemental Table 3: Competing Risk Models for the Association of NTproBNP and cTnI with Outcomes among 503 Hemodialysis Patients of the CHOICE Study**

|  |  |  |  | **Model 1** |  | **Model 2** |  | **Model 3** |  | **Model 4** |  |
| --- | --- | --- | --- | --- | --- | --- | --- | --- | --- | --- | --- |
|  | **Range** | **N (events)** | **Crude**  **IR** | **SHR (95% CI)** | **p** | **SHR (95% CI)** | **p** | **SHR (95% CI)** | **p** | **SHR (95% CI)** | **p** |
| **NTproBNP, pg/ml** | | | | | | | | | | | |
| Continuous ^1^ |  | 503(75) |  | 1.20 (1.11-1.30) | <0.001 | 1.17 (1.08-1.28) | <0.001 | 1.13 (1.03-1.25) | 0.012 | 1.12 (1.01-1.24) | 0.034 |
| Categorical ^2, 4^ |  |  |  |  |  |  |  |  |  |  |  |
| Low Category | 59 - 1710 | 168(13) | 19.0 | Reference |  | Reference |  | Reference |  | Reference |  |
| Mid Category | 1728 - 7269 | 168(22) | 35.3 | 1.46 (0.89-2.41) | 0.135 | 1.47 (0.84-2.58) | 0.181 | 1.33 (0.79-2.24) | 0.284 | 1.22 (0.69-2.15) | 0.503 |
| High Category | 7350 - 273502 | 167(40) | 78.9 | 2.93 (1.68-5.10) | <0.001 | 2.76 (1.51-5.07) | 0.001 | 2.25 (1.12-4.53) | 0.023 | 2.10 (1.00-4.40) | 0.051 |
| *p-trend* |  |  |  | <0.001 |  | <0.001 |  | 0.015 |  | 0.029 |  |
| **Troponin I, ng/mL** | | | | | | | | | | | |
| Continuous ^1^ |  | 503(75) |  | 1.07 (0.95-1.20) | 0.271 | 1.07 (0.94-1.22) | 0.280 | 1.05 (0.91-1.21) | 0.495 | 1.03 (0.87-1.22) | 0.72 |
| Categorical ^2, 3^ |  |  |  |  |  |  |  |  |  |  |  |
| Low Category | <0.015 | 336(41) | 32.8 | Reference |  | Reference |  | Reference |  | Reference |  |
| Mid Category | 0.015 - 0.039 | 85(18) | 57.5 | 1.69 (0.98-2.91) | 0.061 | 1.80 (1.00-3.22) | 0.048 | 1.55 (0.83-2.88) | 0.165 | 1.50 (0.74-3.74) | 0.266 |
| High Category | 0.040 - 3.09 | 82(16) | 64.0 | 1.50 (0.95-2.37) | 0.082 | 1.56 (0.92-2.63) | 0.098 | 1.37 (0.79-2.38) | 0.265 | 1.27 (0.67-2.42) | 0.470 |
| *p-trend* |  |  |  | 0.043 |  | 0.057 |  | 0.209 |  | 0.407 |  |

Abbreviations: Troponin I, cTnI; Sub-Hazard Ratio, SHR; N-terminal pro-brain natriuretic peptide, NTproBNP

Incidence rate per 1000 person-years.

^1^ Sub-Hazard ratio per doubling of the marker; modeled as ln(marker)/ln(2). Modeled using competing-risks regression based on Fine and Gray’s proportional subhazards model. Competing risk for sudden cardiac death is cardiovascular and non-cardiovascular mortality.

^2^ Hazard ratio with the low category as the reference group. Modeled using competing-risks regression based on Fine and Gray’s proportional subhazards model. Competing risk for sudden cardiac death is cardiovascular and non-cardiovascular mortality.

^3^ For cTnI, low category refers those patients with TNI below the limit of detection (<0.015 ng/mL; n=336). Remaining patients are divided into two groups at the median. Mid category refers to the group below median cTnI for those with detectable values (<0.040 ng/mL; n=85) and high category refers to those with values at or above median (≥0.040 ng/mL; n=82).

^4^ For NTproBNP, low, mid and high category refers to the lowest, middle and highest tertiles of NTproBNP.

Model 1: Unadjusted

Model 2: Adjusted for demographics including age, sex and race

Model 3: Adjusted for variables in model 2 + clinical factors including smoking status (ever versus never), Index of Coexistent Disease (ICED) score, diabetes, cardiovascular disease, congestive heart failure, body mass index and systolic blood pressure

Model 4: Adjusted for variables in model 3 + left ventricular hypertrophy, β-blocker use and laboratory tests including hemoglobin, serum albumin, serum potassium, serum bicarbonate, serum corrected calcium and serum phosphate

**Supplemental Table 4: Definitions of Sudden Cardiac Death in Prior and Current Studies**

| **Criterion** | **US Renal Data System Simple Definition** | **Complex Definition-Inpatient (Pun et al 2012** | **Complex Definition-Outpatient (Pun et al 2012)** | **CHOICE Data SCD ascertainment (Parekh et al 2008 and this paper)** |
| --- | --- | --- | --- | --- |
| **Location** | Any | Inpatient | Outpatient | Outpatient, including ED visit or DOA to hospital (excluding hospice) |
| **Data Source** | Form 2746 | Form 2746 and ICD-9-CM codes from Medicare billing data | Form 2746 and ICD-9-CM codes from Medicare billing data | Death certificate and ICD-9 and ICD-10 codes from Medicare billing data |
| **Reported Cause of Death** | Cardiac arrest, cause unknown, or arrhythmia reported on Form 2746 | Primary cause of death due to “cardiac arrhythmia” on form 2746; if supportive claim data, any primary cardiac cause of death | Primary cardiac cause of death on form 2746; if supportive claim data recorded, “unknown” cause of death also accepted | Death occurring out of hospital with the following codes: ICD-9 390–398, 402, or 404–429 or ICD-10 I00–I09, I11, I13, and I20–I51 on death certificates in state vital statistics offices |
| **Inpatient Claims** | Not used | Ventricular fibrillation (427.4) and/or cardiac arrest (427.5) | Ventricular fibrillation (427.4) and/or cardiac arrest (427.5) | Not used |
| **Exclusions** | None | Withdrawal of dialysis; hospice care; primary or secondary cause of death reported as hyperkalemia, septicemia, or malignant disease | Withdrawal of dialysis; hospice care; primary or secondary cause of death reported as hyperkalemia, septicemia, or malignant disease | Hospice; hyperkalemia, sepsis, malignancy listed as secondary cause of death |
